# Confinement and Separation of Benzene from an Azeotropic Mixture Using a Chlorinated B←N Adduct

**DOI:** 10.1021/acs.cgd.4c00125

**Published:** 2024-06-26

**Authors:** Isabella J. Jupiter, Jesus Daniel Loya, Nicholas Lutz, Paulina M. Sittinger, Eric W. Reinheimer, Gonzalo Campillo-Alvarado

**Affiliations:** † Department of Chemistry, Reed College, Portland, Oregon 97202-8199, United States; ‡ Institut für Chemie und Biochemie, Freie Universität Berlin, Arnimallee 22, 14195 Berlin, Germany; § Rigaku Americas Corporation, The Woodlands, Texas 77381, United States

## Abstract

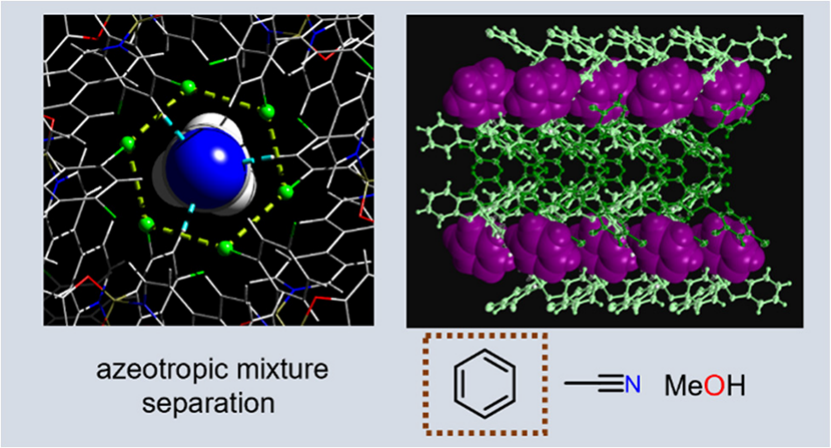

Separations of azeotropic mixtures are typically carried out using energy-demanding processes (e.g., distillation). Here, we report the capacity of a self-assembled chlorinated boronic ester-based adduct to confine acetonitrile and benzene in channels upon crystallization. The solvent confinement occurs via a combination of hydrogen bonding and [π···π] interactions. Quantitative separation of benzene from an azeotropic 1:1 mixture of a benzene/acetonitrile (v/v), and methanol is achieved through crystallization with the chlorinated adduct by complementary [C–H···O] and [C–H···π] interactions. Inclusion behavior is rationalized by molecular modeling and crystallographic analysis. The chlorinated boronic ester adduct shows the potential of modularity via isosteric substitution for the separation of challenging chemical mixtures (e.g., azeotropes).

## Abstract 2

We demonstrate the use of a chlorinated boronic ester-based adduct to separate benzene from an azeotropic mixture of benzene/acetonitrile/methanol by crystallization. Chemical separations of azeotropes (i.e., mixtures that exhibit the same concentration in the vapor and liquid phases) are fundamentally challenging and require alternative methods to traditional distillation. We highlight the opportunities of highly modular boron adducts to provide control over chemical separations of mixtures via selective crystallization.

Efficient chemical separations of petrochemicals and small molecules are industrially relevant due to the need for pure chemical feedstock for plastics, drugs, and fuel.^1^ In the U.S., chemical separations carried out by traditional methods (e.g., distillation) account for 10–15% of the total energy consumption.^2^ The problem is exacerbated when chemical mixtures exhibit complex phenomena. The formation of azeotropes in mixtures (i.e., the vapor phase has the same composition as a liquid phase) is a relevant example that requires the addition of entrainers to ensure efficient azeotropic distillations.^3^ Consequently, explorations of sustainable, green, and less-energy-demanding alternatives for complex chemical separations (e.g., metal–organic or covalent-organic frameworks)^1,4^ are a critical demand for industry and academia.^1,5^

Our group and others have employed boronic ester coordination with pyridines (B←N)^6^ to generate H-shaped^7^ and T-shaped^8^ adducts. The adducts have enabled the confinement and separation of petrochemicals,^9^ and the design of electronic^10^ and dynamic materials.^11^ Our design has exploited the generation of electron-deficient surfaces resulting from coordinated pyridyl linkers to boronic esters and aided by additional noncovalent interactions (e.g., [C–H···F]) with 2,4-difluorophenylboronic acid (**F-ba**).^7^ To modulate properties of B←N adducts, we envisage isosteric substitution (i.e., replacement of a functional group with another of similar electronic structure)^12^ of boronic ester adducts (e.g., replacing -F for -Cl in the boronic acid) can result in diverse selectivities and confinement modes that could promote the separation of challenging chemical mixtures (e.g., azeotropes). Isosteric substitution has been used to modulate π-stacking modes in organic semiconductors^13^ to promote photoreactivity,^14^ and activate molecular motion^15^ in the solid state.

Here, we demonstrate the use of a chlorinated boronic ester adduct (**Cl-1**) to confine and separate acetonitrile (**MeCN**) and benzene (**ben**). The boron adduct is formed by self-assembly of 2,4-dichlorophenylboronic acid (**Cl-ba**), catechol (**cat**), and 4,4′-bipyridine (**bpy**) in **MeCN** or **ben** (Scheme 1a). Confinement of **MeCN** is supported by the generation of weak halogen bonding (i.e., [Cl···Cl]) between the adducts, which was absent in fluorinated systems, in addition to [C–H···N] contacts). Confinement of **ben** relies on [C–H···O] and [C–H···π] contacts. In contrast to previous studies, the guests sit on the boronic ester periphery of the B←N adduct rather than on the electron-deficient surface of **bpy**. The resulting solvent confinement mode results in the formation of “side” pockets instead of enclosed pockets as previously observed (Scheme 1b,c).^7^ Applicability of **Cl-1** for the separation of **ben** and **MeCN** was demonstrated by crystallization (i.e., selective uptake of **ben** from an isovolumetric mixture) (Scheme 1d). Rationale for the separation is provided by a combination of crystallographic analysis with molecular calculations performed using the Hartree–Fock method (HF/3-21G basis set). To our knowledge, our study represents the first example of an azeotropic separation (i.e., acetonitrile/benzene/methanol) carried out via crystallization using a supramolecular host.

**Scheme 1:**
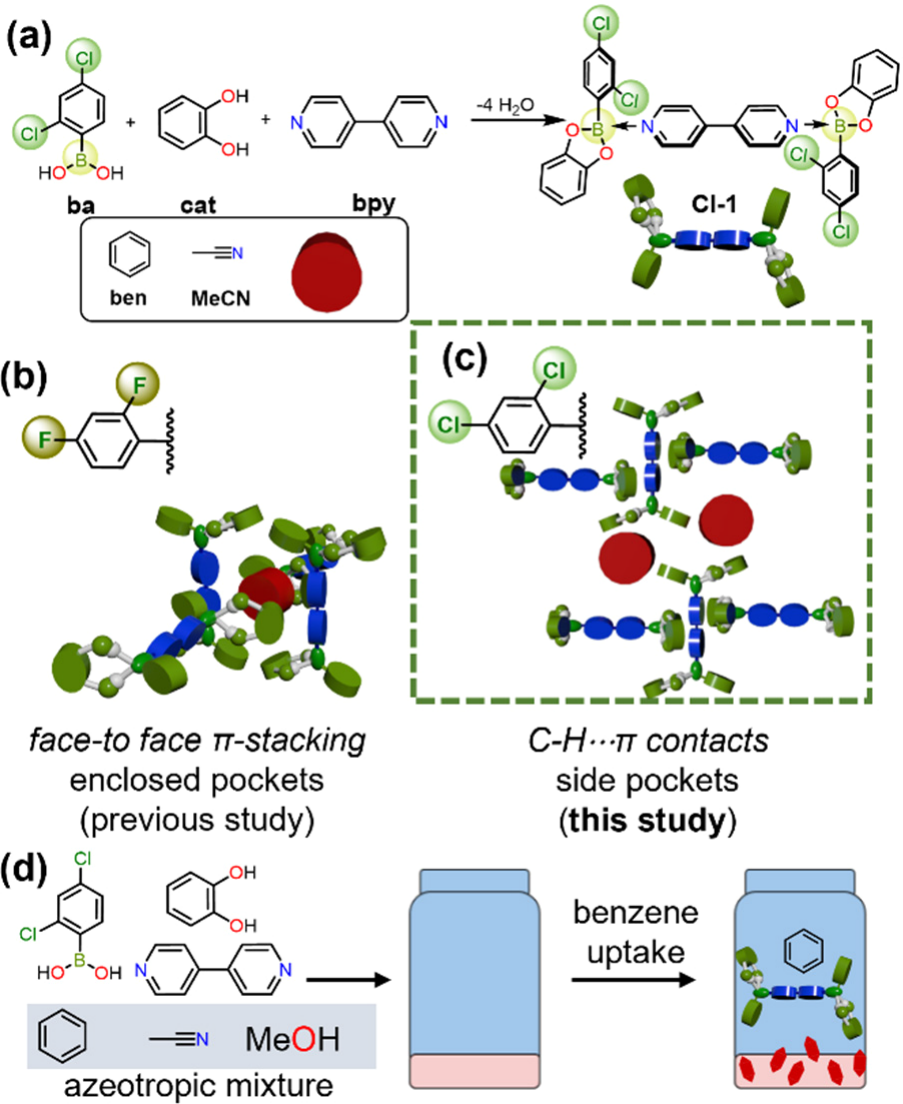
Design and Application of Chlorinated Adduct **Cl-1**: (a) Self-Assembly of **Cl-1**; Confinement Modes in (b) Previous Studies^7^ and (c) This Study; and (d) Separation of Benzene from an Azeotropic Mixture with Adduct **Cl-1** via Crystallization

To evaluate the modularity of B←N adducts, **Cl-ba** (12.2 mg, 0.0639 mmol) was combined with **cat** (7.04 mg, 0.0639 mmol) and **bpy** (5.0 mg, 0.0320 mmol) in **MeCN** (3 mL) with dropwise addition of methanol (ca. 0.5 mL). The vial was gently heated until the solution was clear. After 3 days of slow evaporation, single crystals of **Cl-1**⊃**MeCN** formed as yellow blades. The stoichiometry of the crystals was confirmed by ^1^H nuclear magnetic resonance (NMR) spectroscopy (See SI).

A single crystal X-ray diffraction (SCXRD) analysis of **Cl-1**⊃**MeCN** revealed the system to crystallize in the trigonal space group *R*-3. The asymmetric unit consists of half a molecule of **1** and one molecule of **MeCN**. Linker **bpy** is coordinated to two phenylboronic acid catechol ester (**be**) units through a B←N bond (1.671 Å), forming a discrete H-shaped adduct where phenyl rings are in *anti*-conformation (Figure 1a). The pyridyl rings are effectively coplanar. The calculated tetrahedral character of four-coordinate boron (*THC* = 69.1%)^16^ is slightly smaller than fluorinated H-shaped B←N adducts (∼72%), indicating a weaker interaction.^7^ In the system, adducts **Cl-1** assemble into tapes in the *ac*-plane sustained by face-to-face [π···π] embrace between adjacent **bpy** and the boronic esters (Figure 1b). Notably, **MeCN** is confined in hexagonal pockets through hydrogen bonds ([C–H···N] = 2.785 Å) with the chlorinated phenyl ring of **Cl-1**. Chlorine atoms aggregate in a regular hexagons via [Cl···Cl] contacts (3.320 Å) through the *c*-axis, highlighting the structure-forming ability of the interactions.^17^ The adduct aggregation is additionally supported by edge-to-face [C–H···π] interactions between hosts (C–H···centroid(catecholate) = 2.801 Å). The **MeCN** guests occupy 11.3% (contact surface analysis) of the unit cell volume and are regularly situated within the side pockets (Figure 1c,d). Numerous attempts to confine **MeCN** with the analogous fluorinated adduct (**F-1**) were unsuccessful.^7^

**Figure 1:**
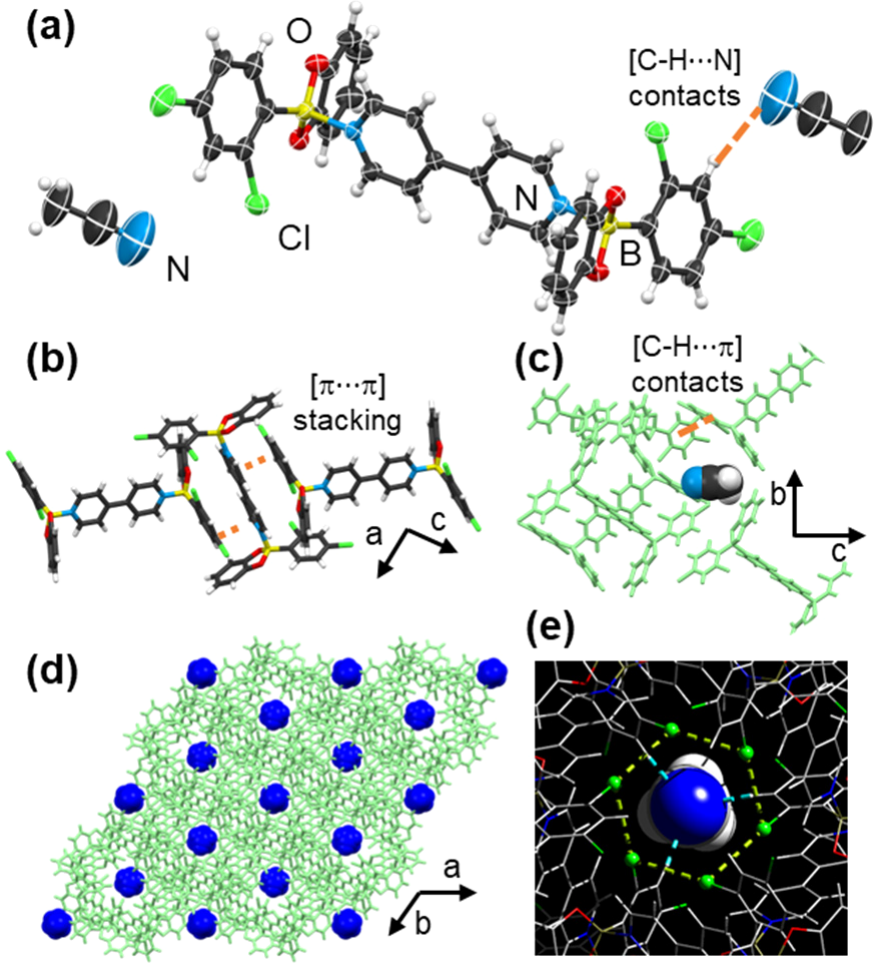
Single crystal X-ray structure of **Cl-1**⊃**MeCN**: (a) Molecular unit of **Cl-1** interacting with MeCN via [C–H···N] contacts. (b) Face-to-face π-stacking between adjacent **bpy** and **be** molecules. (c) Edge-to-face [C–H···π] contacts between **Cl-1** units. (d) Formation of side pockets in the *ab*-plane. (e) Hexagonal architecture of pockets via short [Cl···Cl] contacts.

We have determined that the **Cl-1** adduct can be exploited to separate **ben** from **MeCN**, and methanol. Because of the similar boiling points of **MeCN** and **ben** (81.6 and 80.1 °C, 1 atm)^18^ and azeotrope formation, the separation of the azeotropic mixture is fundamentally challenging and relevant for industry.^19^ Current separation methods employ energy-demanding distillation with entrainers to increase the relative volatility of compounds and improve separation.^20^ When the starting materials **Cl-ba**, **cat**, and **bpy** (using the same for **Cl-1**⊃**MeCN**) were dissolved in a 1:1 **MeCN**/**ben** solution (3 mL, v/v) and ca. 0.5 mL of methanol (used to facilitate complete dissolution), single crystals in the form of orange blocks precipitated after a period of 1 day. While we note the role of methanol to be a solubilizing agent, azeotropes of methanol/**ben** and methanol/**MeCN** are likely present as ternary azeotropic system.^19^ Remarkably, filtered single crystals crystallized with **ben** quantitatively as the only solvent confined, as indicated by ^1^H NMR spectroscopy (see SI). Crystals of pure **Cl-1** were not observed in the crystallization vial. The performance is comparable to existing separation methods for azeotropic separations using triple-column pressure-swing distillation^19^ or entrainers.^20^ Partial recovery of the **Cl-1** host was enabled by heating the **Cl-1**⊃**ben** crystals at 100 °C for 24 h. ^1^H NMR spectroscopy confirmed **ben** desolvation of 14% after heating at 100 °C for 5 min. Additional 15 min of heating afforded 45% **ben** desolvation (see SI). Prolonged heating resulted in sample decomposition. We envisage the partial recovery of **Cl-1** will inspire the design of methods to enable full recovery of hosts after guest uptake from azeotropic mixtures, ensuring sustainability and recyclability of separation processes.^21^

Structural determination by SCXRD revealed the components of **Cl-1**⊃**ben** to crystallize in the monoclinic space group *C*2/*c* (Table 1). The stoichiometry of the crystals was confirmed by ^1^H NMR spectroscopy (see SI). The asymmetric unit comprises two one-halves of **Cl-1** (i.e., **1a** and **1b**) and half a molecule of **ben** (Figure 2a). The [B←N] bond distances of **1a** and **1b** (1.667 and 1.646 Å, respectively) and calculated *THC* (73.9 and 77.1%, respectively) are comparable to those of previously reported H-type adducts.^7^ It is noteworthy that the twist angles of pyridyl rings in **1a** and **1b** are 64.2° and 0°, respectively. We hypothesize the twisted **bpy** in **1a** is due to the loss of efficient conjugation of the π-cloud in the molecule to favor face-to-face [π···π]-stacking of individual pyridine rings with **be** motifs of adjacent **Cl-1** molecules, and to maximize edge-to-face [C–H···π] interactions between the pyridyl and dichlorophenyl rings.^22^ Intermolecular π-stacking interactions between adducts generate side pockets along the *b*-axis in the crystal that contain **ben** molecules (19.5% of unit cell volume, contact surface analysis) (Figure 2**b,c**). Molecules of **ben** are supported by [C–H···O] and [C–H···π] contacts with the catechol and phenyl ring moieties, respectively, in the **be** (Figure 2d). Confinement of **ben** with **Cl-1** (Figure 2e) differs from the analogous fluorinated B–N adduct (**F-1**), which relies on the formation of enclosed pockets primarily by face-to-face [π···π] stacking with the **bpy** linker and additional edge-to-face [C–H···π] stacking, and [C–H···F] contacts.^7^

**Table 1:** Summary of Crystallographic Data for **Cl-1**⊃**MeCN**, **Cl-1**⊃**ben**, **Cl-1**, and **F-1**

**crystal data**^a^	**Cl-1**⊃**MeCN**	**Cl-1**⊃**ben**	**Cl-1**	**F-1**
chemical formula	3(C_34_H_22_B_2_ Cl_4_N_2_O_4_)·2(C_2_H_3_N)	2(C_17_H_11_BCl_2_NO_2_)·0.5(C_6_H_6_)	C_34_H_22_B_2_Cl_4_N_2_O_4_	C_34_H_22_B_2_F_4_ N_2_O_4_
MW (g mol^–1^)	2139.97	725.01	685.95	620.15
space group	*R*-3	*C*2/*c*	*P*2_1_/*c*	*P*2_1_/*n*
*a* (Å)	19.5553(12)	24.8867(10)	9.4842(5)	9.3110(9)
*b* (Å)	19.5553(12)	10.3952(5)	12.8576(7)	13.0637(18)
*c* (Å)	22.4722(16)	26.6246(9)	13.1489(5)	13.1643(17)
α (deg)	90	90	90	90
β (deg)	90	98.089(4)	95.932(4)	109.440(12)
γ (deg)	120	90	90	90
V (Å^3^)	7442.3(11)	6819.3(5)	1594.84(14)	1510.0(3)
Z	3	8	2	2
μ (mm^–1^)	0.403	0.391	0.414	0.105
ρ_calcd_ (g cm^–3^)	1.432	1.412	1.428	1.364
*R*_1_^b^,^c^	0.0894	0.0479	0.0478	0.0648
w*R*_2_^d^,^e^	0.2253	0.1075	0.1191	0.1683
CCDC	2327025	2327023	2327022	2327024

a λ_MoKα_ = 0.71073 Å.

b *F*_0_ > 2σ(*F*_0_).

c *R*_1_ = ∑|*F*_0_| – |F_c_|/∑|*F*_0_|.

d All data.

e *wR*_2_= [∑*w*(*F*_0_^2^ – *F*_c_^2^)^2^/∑*w*(*F*_0_^2^)^2^]^1/2^.

**Figure 2:**
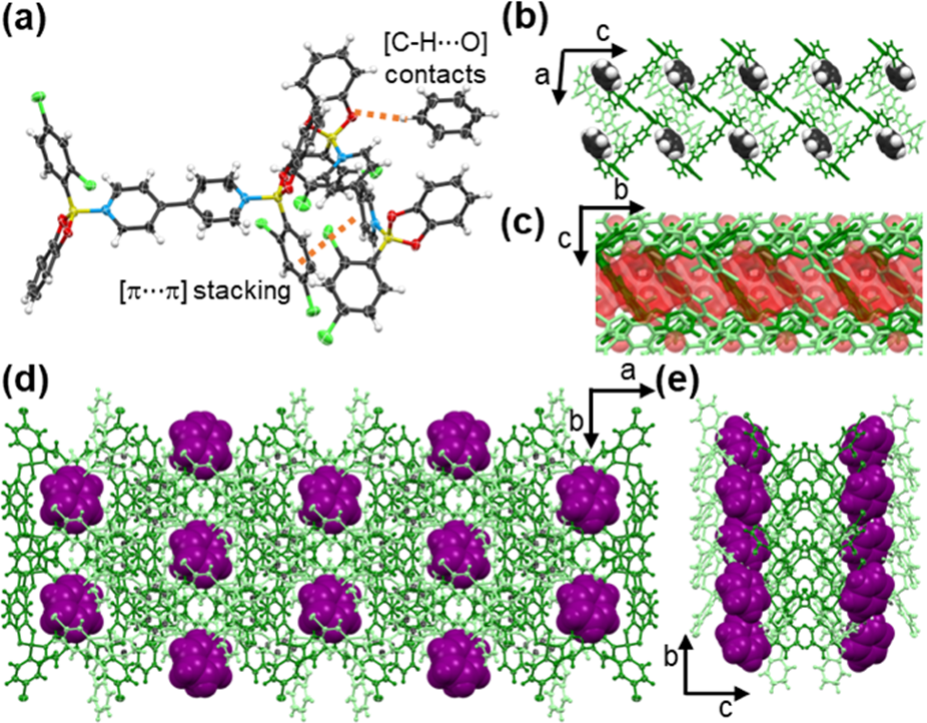
Single crystal X-ray structure of **Cl-1**⊃**ben**: (a) Molecular unit of **Cl-1** interacting with **ben** via [C–H···O] contacts. (b) Encapsulated **ben** molecules in the periphery of the **be** motif. (c) Channel volume (highlighted in red). (d) Inclusion of **ben** molecules in side pockets in the *ab*-plane. (e) Side pockets formed along the *b*-axis.

During the course of our studies, single crystals of pure host **Cl-1** (i.e., apohost) in the form of yellow blocks were harvested in minor amounts from the vial containing **Cl-1**⊃**MeCN**. SCXRD analysis revealed the apohost to self-assemble in the monoclinic space group *P*2_1_/*c* (Figure 3a). The asymmetric unit contains one-half of the **Cl-1** adduct with [B←N] bond distance and *THC* of 1.657 Å and 75.3%, respectively. The values are comparable to the solvated **Cl-1**⊃**ben** adduct. The bipyridyl rings in **bpy** are effectively coplanar. In the system, the **Cl-1** adducts self-assemble into tapes that run along the *c-*axis. The tapes are sustained by a combination of [C–Cl···O], [C–H···π], and phenyl embraces generated by face-to-face [π···π] interactions (Figure 3b). Notably, the crystal structure has spherical voids that account for 2.6% of the unit cell volume (40.7 Å^3^). The observation supports the solvate-forming propensity of **Cl-1** to decrease void space and lead to more efficient packing (Figure 3c).^23^ Notably, **Cl-1** does not exhibit short [Cl···Cl] contacts, which are present in both solvated systems. An isoskeletal structure of **F-1** was isolated during our studies with 2,4-difluorophenylboronic acid. The structure was deemed a polymorph of **F-1** that exhibits an inversion center between the pyridyl rings of **bpy** (i.e., rings are coplanar), which is absent in the previously reported structure (see SI for structural analysis).^7^

**Figure 3:**
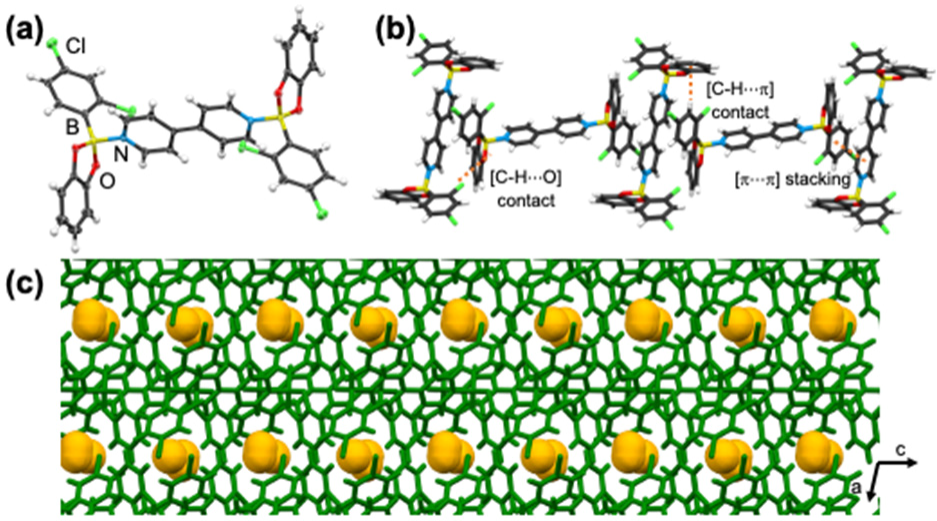
Single crystal X-ray structure of **Cl-1**: (a) Molecular unit of **Cl-1**. (b) Tapes of adjacent **Cl-1** adducts supported by [C–Cl···O], [C–H···π], and [π···π] contacts. (c) Voids formed along the *c*-axis.

Molecular coordinates obtained from single crystals from **Cl-1** and the **F-1** polymorph enabled us to perform Hirshfeld surface analysis^24^ and molecular modeling to provide a rationale for the solvent inclusion and selectivity of **Cl-1** (Figure 4a,b). For the apohosts, **F-1** showed the presence of minimal F···F interactions, while **Cl-1** showed no [Cl···Cl] interactions. Upon inclusion with **ben** and **MeCN,** there was an increase in [Cl···Cl] interactions in both inclusion complexes with **Cl-1**. Specifically, the percentage of [Cl···Cl] interactions in **Cl-1**⊃**ben** and **Cl-1**⊃**MeCN** increased to 1.6% and 4.2%, respectively (Figure 4c). Halogen bonding aided in the aggregation of the adducts. In the case of **Cl-1**⊃**MeCN**, the adduct formed hexagonal-shaped pockets sustained by [Cl⊃Cl] interactions. For **Cl-1**⊃**ben,** while the increase of [Cl···Cl] was minimal, the combination with [C–H···π], [C–H···O], and [π···π] contacts supported **ben** confinement in side pockets along the crystallographic *b*-axis (Table S5), and enhanced selectivity over **MeCN** and methanol when cocrystallized in an azeotropic mixture with **Cl-1**. The formation of halogen bonding interactions in **Cl-1**⊃**ben** and **Cl-1**⊃**MeCN** is in agreement with the increase in the σ-hole and a larger negative belt surface area observed in electrostatic potential maps from molecular modeling, as shown in calculations performed using the Hartree–Fock method (HF/3-21G basis set) of **Cl-1**, which is larger than surfaces generated in **F-1**. Specifically, σ-holes in Cl1 and Cl2 from **Cl-1** were calculated as ca. 28 and 66 kJ/mol, respectively, while both F1 and F2 from **F-1** were ca. −129 kJ/mol, indicating a more effective surface for halogen bonding in **Cl-1** (Figure 4d), which is in agreement with the formation of [Cl···Cl] interactions in **Cl-1**⊃**MeCN**.

**Figure 4:**
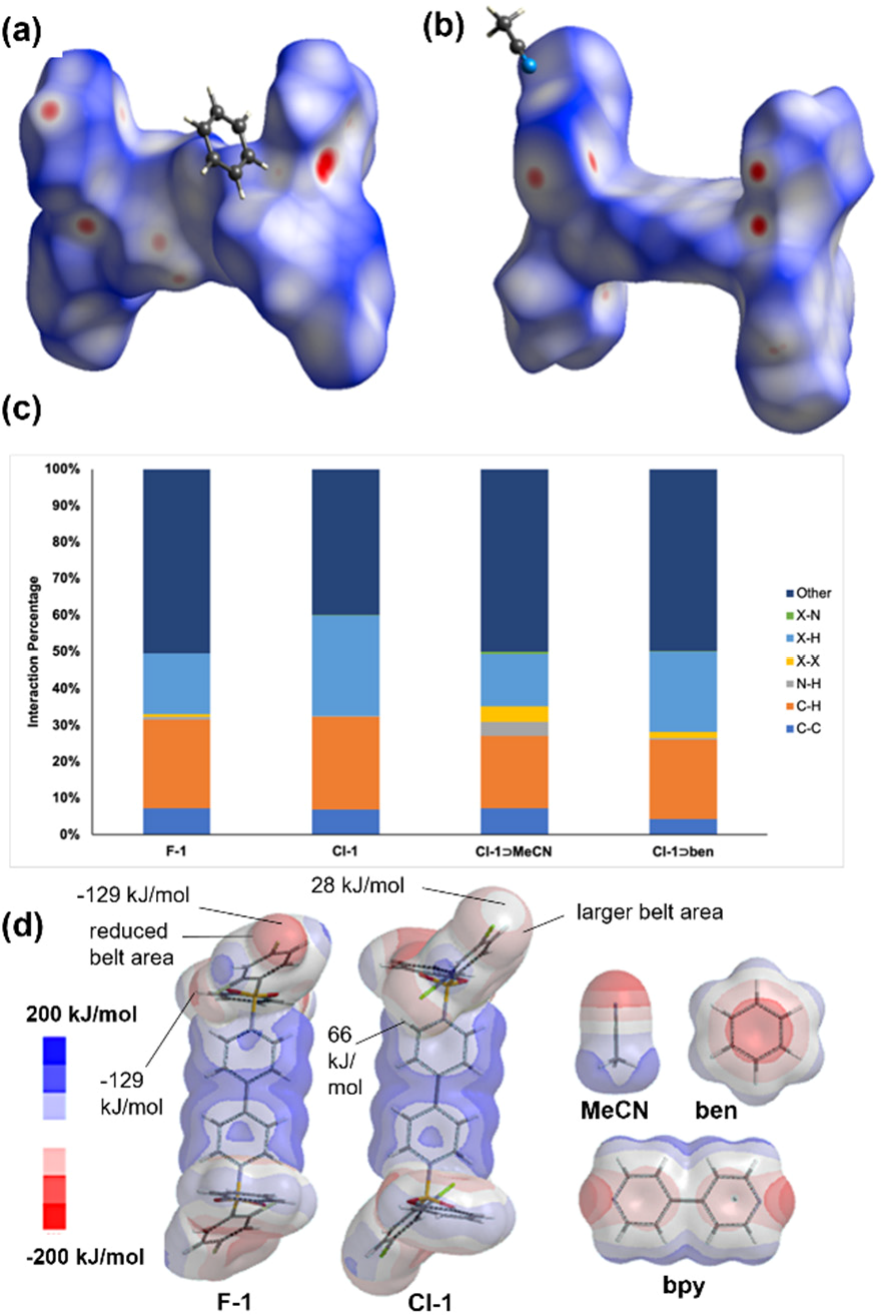
Hirshfeld surface analysis maps of (a) **Cl-1**⊃**MeCN** and (b) **Cl-1**⊃**ben**. (c) Selected projection interaction percentages of the reported structures. (d) Electrostatic potential maps of **F-1**, **Cl-1**, **ben**, **MeCN**, and **bpy**.

In summary, we have highlighted the potential of confinement modularity of boronic ester-based adducts using isosteric substitution (i.e., replacing -F with -Cl). Specifically, we demonstrated that by installing -Cl atoms to adduct **Cl-1**, selective uptake and separation of benzene from an azeotropic mixture of benzene/acetonitrile/methanol was achieved. We envisage the highly modular nature of boron adducts can result in the separation of additional challenging mixtures,^2^ and serve as a proof-of-concept to engineer alternatives to energy-demanding distillation methods in industry.^21^

## Supporting Information Available

Experimental conditions and additional data for single crystal X-ray diffraction, powder X-ray diffraction, molecular modeling, and ^1^H and ^19^F nuclear magnetic resonance (PDF)
