# Supplementary material for: Confinement and Separation of Benzene from an Azeotropic Mixture Using a Chlorinated B←N Adduct
Source: Cryst Growth Des. 2024 Jun 26;24(14):5883–8. doi: 10.1021/acs.cgd.4c00125 (PMC11261595; doi:10.1021/acs.cgd.4c00125)
Supplement: Supplementary file 1 — cg4c00125_si_001.pdf [file cg4c00125_si_001.pdf]

Supporting Information (SI)

Confinement and Separation of Benzene from an Azeotropic Mixture Using a Chlorinated B←N Adduct

Isabella J. Jupiter,[a,†] Jesus Daniel Loya, [a,†] Nicholas Lutz,[a] Paulina M. Sittinger,[a,b] Eric W. Reinheimer,[c] and Gonzalo Campillo-Alvarado [a,]*

[a] Department of Chemistry, Reed College, Portland, OR 97202-8199 USA
[b] Institut für Chemie und Biochemie, Freie Universität Berlin, Arnimallee 22, 14195 Berlin, Germany
[c] Rigaku Americas Corporation, The Woodlands, Texas 77381, USA
† These two authors contributed equally

Supplementary Information:

S1) Experimental information

S2) Single-crystal X-ray data

S3) NMR spectral data

S4) Powder X-ray data

S5) Energy Framework Calculations

S1. Experimental information

Materials:

Phenylboronic acid (**Cl-ba**) and 4-4'-bipyridine (**bpy**) were obtained from AmBeed, and catechol (**cat**) was obtained from Combi-Blocks. Benzene (**ben**) and chloroform were obtained from TCI, and acetonitrile (**MeCN**) from Sigma Aldrich. All materials were used without further purification.

Solvated adduct **Cl-1⊃MeCN** was generated by dissolving 5 mg (0.0320 mmol) of **bpy**, 7.04 mg (0.0639 mmol) of **cat**, and 12.2 mg (0.0639 mmol) of **Cl-ba** in **MeCN** (3 mL) and methanol (0.5 mL), assisted with heat and sonication until the solution became clear. Crystals suitable for single crystal X-ray diffraction (SCXRD) formed after a period of 3 days upon slow solvent evaporation. Yield: 22 mg (47%)

Solvated adduct **Cl-1⊃ben** was generated by dissolving 5 mg (0.0320 mmol) of **bpy**, 7.04 mg (0.0639 mmol) of **cat**, and 12.2 mg (0.0639 mmol) of **Cl-ba** in **ben** (3 mL), assisted with heat, sonication, and dropwise addition of methanol (ca. 0.5 mL) until the solution became clear. Single crystals suitable for SCXRD formed after a period of 1 day upon slow solvent evaporation. Yield: 26 mg (53%)

Chemical separation studies using **Cl-1** were performed by 5 mg (0.0320 mmol) of **bpy**, 7.04 mg (0.0639 mmol) of **cat**, and 12.2 mg (0.0639 mmol) of **Cl-ba** in a 1:1 (v/v) binary mixture of the solvents **MeCN/ben** (3 mL), assisted with heat, sonication, and dropwise addition of methanol (ca. 0.5 mL) until the solution became clear. Single crystals suitable for SCXRD formed after a period of 1 day upon slow solvent evaporation in a vial with the cap on to prevent solvent evaporation. Yield: 31 mg (61%) The resulting crystals were rapidly filtered, dried, and analyzed by ^{1}H NMR spectroscopy.

Instruments and methods:

Single crystal X-ray diffraction (SCXRD) data was collected on a Rigaku XtaLAB Mini II diffractometer with a CCD area detector (λMoKα = 0.71073 Å, monochromator: graphite) equipped with an Oxford Cryostream low temperature device. Experiments were conducted at 100 K with a range of 2θ = 3-62°. The collected data was refined with CrysAlisPro through standard data reduction and background corrections (analytical for **F-1**, and multi-scan for **Cl-1⊃MeCN**, **Cl-1⊃ben**, **Cl-1**). Crystals were mounted in Paratone oil on a Mitegen magnetic mount. Structure solution and refinement were performed using SHELXT[1] and SHELXL,[2] respectively, within the Olex2[3] graphical user interface. Non-hydrogen atoms were refined anisotropically, and hydrogen atoms were placed in geometrically calculated positions using a riding model. Crystal structures were generated using Mercury. Metrics were calculated using Olex2 from the .res files using shelxl matrices. ^{1}H NMR spectra were recorded on a Bruker AV400 and on a Bruker Ascend Evo spectrometer with chloroform-d, DMSO-d6 and TMS as internal standards. All NMR data was processed with the Mnova suite.

S2. Single-crystal X-ray data.

Table S1. Crystallographic parameters for **Cl-1⊃MeCN**

Compound name	Cl-1⊃MeCN
Empirical formula	$3(C_{34}H_{22}B_2 Cl_4N_2O_4), 2(C_2H_3N)$
Formula weight	2139.97
Temperature/K	100.00(10)
Crystal system	trigonal
Space group	R-3
a/Å	19.5553(12)
b/Å	19.5553(12)
c/Å	22.4722(16)
α/°	90
β/°	90
γ/°	120
Volume/Å^3	7442.3(11)
Z	3
ρ_{calc}g/cm^3	1.432
μ/mm^{-1}	0.403
F(000)	3282.0
Crystal size/mm^3	0.4 × 0.19 × 0.07
Radiation	Mo Kα (λ = 0.71073)
2Θ range for data collection/°	4.35 to 50.244
Index ranges	$-23 \le h \le 23, -23 \le k \le 23, -26 \le l \le 26$
Reflections collected	34371
Independent reflections	2966 [R_{int} = 0.1176, R_{sigma} = 0.0655]
Data/restraints/parameters	2966/53/246
Goodness-of-fit on F^2	1.083
Final R indexes [I>=2σ (I)]	R_1 = 0.0894, wR_2 = 0.2086
Final R indexes [all data]	R_1 = 0.1220, wR_2 = 0.2253
CDCC Identification Code	2327025

Table S2. Crystallographic parameters for **Cl-1⊃ben**

**Compound name**	**Cl-1⊃ben**
**Empirical formula**	$2(C_{17}H_{11}BCl_2NO_2),0.5(C_6H_6)$
**Formula weight**	725.01
**Temperature/K**	100.00(10)
**Crystal system**	monoclinic
**Space group**	C2/c
**a/Å**	24.8867(10)
**b/Å**	10.3952(5)
**c/Å**	26.6246(9)
**α/°**	90
**β/°**	98.089(4)
**γ/°**	90
**Volume/Å^3**	6819.3(5)
**Z**	8
**ρ_{calc}g/cm^3**	1.412
**μ/mm^{-1}**	0.391
**F(000)**	2968.0
**Crystal size/mm^3**	0.336 × 0.209 × 0.135
**Radiation**	Mo Kα (λ = 0.71073)
**2Θ range for data collection/°**	4.196 to 52.744
**Index ranges**	-31 ≤ h ≤ 31, -12 ≤ k ≤ 12, -33 ≤ l ≤ 33
**Reflections collected**	30736
**Independent reflections**	6954 [R_{int} = 0.0431, R_{sigma} = 0.0480]
**Data/restraints/parameters**	6954/6/448
**Goodness-of-fit on F^2**	1.025
**Final R indexes [I>=2σ (I)]**	R_1 = 0.0479, wR_2 = 0.0967
**Final R indexes [all data]**	R_1 = 0.0794, wR_2 = 0.1075
**CDCC Identification Code**	2327023

Table S3. Crystallographic parameters for **Cl-1**

**Compound name**	**Cl-1**
**Empirical formula**	$C_{34}H_{22}B_2Cl_4N_2O_4$
**Formula weight**	685.95
**Temperature/K**	100.00(10)
**Crystal system**	monoclinic
**Space group**	$P2_1/c$
**a/Å**	9.4842(5)
**b/Å**	12.8576(7)
**c/Å**	13.1489(5)
**α/°**	90
**β/°**	95.932(4)
**γ/°**	90
**Volume/Å³**	1594.84(14)
**Z**	2
**ρ_{calc}g/cm³**	1.428
**μ/mm⁻¹**	0.414
**F(000)**	700.0
**Crystal size/mm³**	0.472 × 0.199 × 0.065
**Radiation**	Mo Kα (λ = 0.71073)
**2Θ range for data collection/°**	4.318 to 56.56
**Index ranges**	-12 ≤ h ≤ 11, -16 ≤ k ≤ 17, -17 ≤ l ≤ 17
**Reflections collected**	12065
**Independent reflections**	3926 [R_{int} = 0.0620, R_{sigma} = 0.0765]
**Data/restraints/parameters**	3926/0/208
**Goodness-of-fit on F^2**	1.030
**Final R indexes [I>=2σ (I)]**	R_1 = 0.0478, wR_2 = 0.1089
**Final R indexes [all data]**	R_1 = 0.0747, wR_2 = 0.1191
**CDCC Identification Code**	2327022

Table S4. Crystallographic parameters for **F-1**.

**Compound name**	**F-1**
**Empirical formula**	$C_{34}H_{22}B_2F_4N_2O_4$
**Formula weight**	620.15
**Temperature/K**	100.15
**Crystal system**	monoclinic
**Space group**	$P2_1/n$
**a/Å**	9.3110(9)
**b/Å**	13.0637(18)
**c/Å**	13.1643(17)
**α/°**	90
**β/°**	109.440(12)
**γ/°**	90
**Volume/Å^3**	1510.0(3)
**Z**	2
**ρ_{calc}g/cm^3**	1.364
**μ/mm^{-1}**	0.105
**F(000)**	636.0
**Crystal size/mm^3**	0.53 × 0.43 × 0.12
**Radiation**	Mo Kα (λ = 0.71073)
**2Θ range for data collection/°**	4.526 to 50.242
**Index ranges**	-10 ≤ h ≤ 11, -15 ≤ k ≤ 15, -15 ≤ l ≤ 15
**Reflections collected**	10710
**Independent reflections**	2691 [R_{int} = 0.0651, R_{sigma} = 0.0905]
**Data/restraints/parameters**	2691/108/250
**Goodness-of-fit on F^2**	1.022
**Final R indexes [I>=2σ (I)]**	R_1 = 0.0648, wR_2 = 0.1403
**Final R indexes [all data]**	R_1 = 0.1377, wR_2 = 0.1683
**CDCC Identification Code**	2327024

Table S5. Selected intermolecular interactions in crystals.

Crystal/ parameter	*d*(X···N) (Å)	*d*(X···H) (Å)	*d*(X···X) (Å)	*d*(N···H) (Å)	*d*(C-H···π) (Å)	symmetry code
**Cl-1⊃MeCN**	3.796(4)[1]	-	-	-	-	
	-	3.1954(19)[2]	-	-	-	(2/3-Y+X, -1/3+X, 4/3-Z)
	-	-	3.320(2)[3]	-	-	(-1/3+Y,1/3-X+Y, 4/3-Z)
	-	-	-	3.463(4)[4]	-	(1-Y,1+X-Y, +Z)
	-	-	-	-	2.801(2)[5]	(1/3-Y+X, -1/3+X, 5/3-Z)
**Cl-1⊃ben**	4.4133(19)[6]	-	-	-	-	(+X, 1-Y, -1/2+Z)
	-	3.0865(7)[7]	-	-	-	
	-	-	3.589(2)[8]	-	-	(3/2-X, 3/2-Y, -Z)
	-	-	-	3.4400(19)[9]	-	(1-X, +Y, 1/2+Z)
	-	-	-	-	2.6234(11)[10]	(-1/2+X, -1/2+Y, +Z)
**Cl-1**	4.0720(16)[11]	-	-	-	-	(1-X, -1/2+Y, 3/2-Z)
	-	2.9605(6)[12]	-	-	-	(1-X, -1/2+Y, 3/2-Z)
	-	-	-	-	-	
	-	-	-	3.80908[13]	-	(+X, 3/2-Y, 1/2+Z)
	-	-	-	-	3.0824(9)[14]	(1-X, -1/2+Y, 3/2-Z)
**F-1**	-	-	-	-	-	
	-	2.672(2)[15]	-	-	-	(-1+X, +Y, +Z)
	-	-	2.816(4)[16]	-	-	(-X, -Y, 1-Z)
	-	-	-	3.580(3)[17]	-	(1/2-X, 1/2+Y, 1/2-Z)
	-	-	-	-	2.9441(15)[18]	(-1+X, +Y, +Z)

[1]Cl2A···N1, [2]Cl1A···N2, [3]Cl2A···Cl2A, [4]N1···H5A , [5]C5A-H5A···π (centroid: C7, C8, C9, C10, C11,C12), [6]Cl2···N00A, [7]Cl4···H16, [8]Cl1···Cl1, [9]N009···H23, [10]C34B-H34B···π(centroid: C7, C8, C9, C10, C11, C12), [11]Cl2···N1, [12] Cl2···H8, [13] N1···H6, [14]C3-H3···π(centroid: C7, C8, C9, C10, C11, C12), [15]F1···H16, [16]F1···F1, [17]N1···H10A, [18]C10-H10···π(centroid: C1, C2, C3, C4, C5, C6)

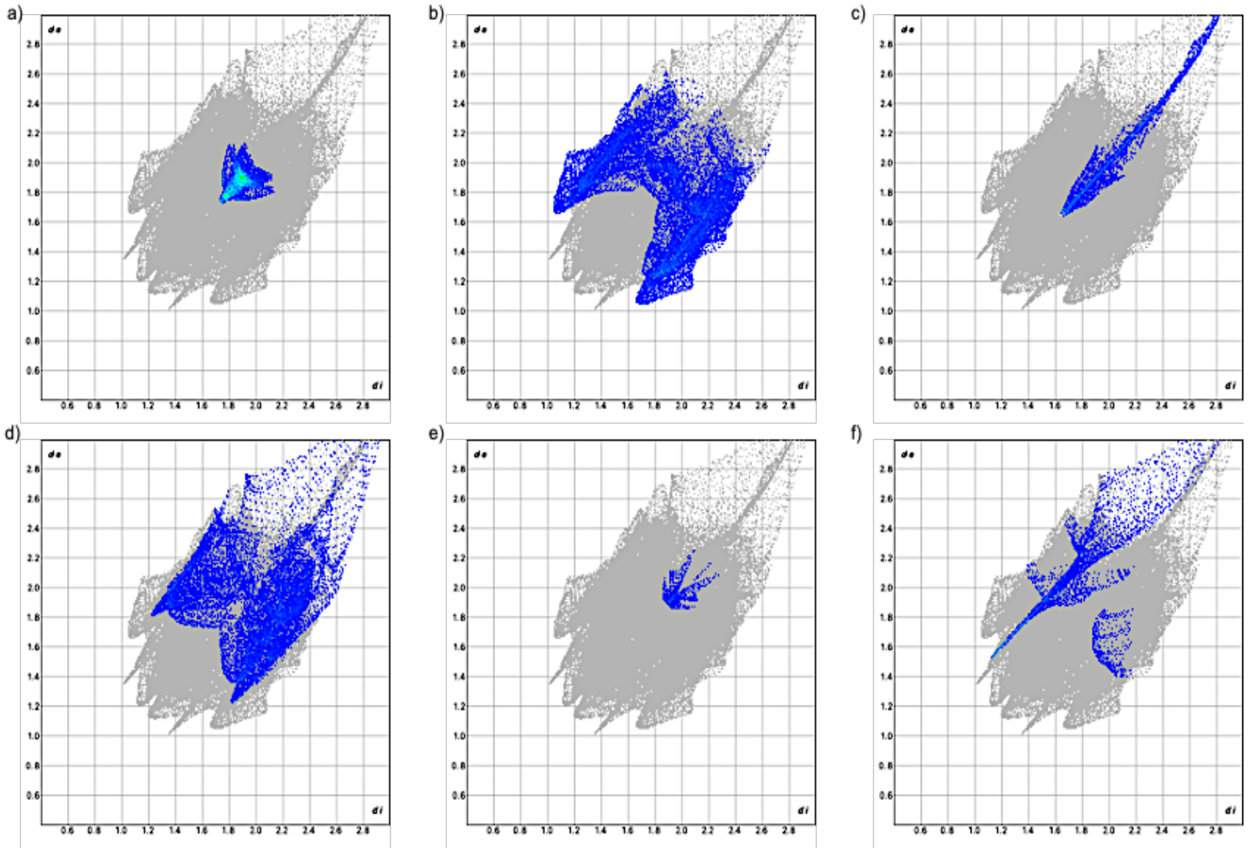

Figure S1. Selected Hirshfeld fingerprint projection interactions for **Cl-1⊃MeCN** structure: (a) C-C interactions, (b) C-H interactions, (c) Cl-Cl interactions, (d) Cl-H interactions, (e) Cl-N interactions, and (f) N-H interactions.

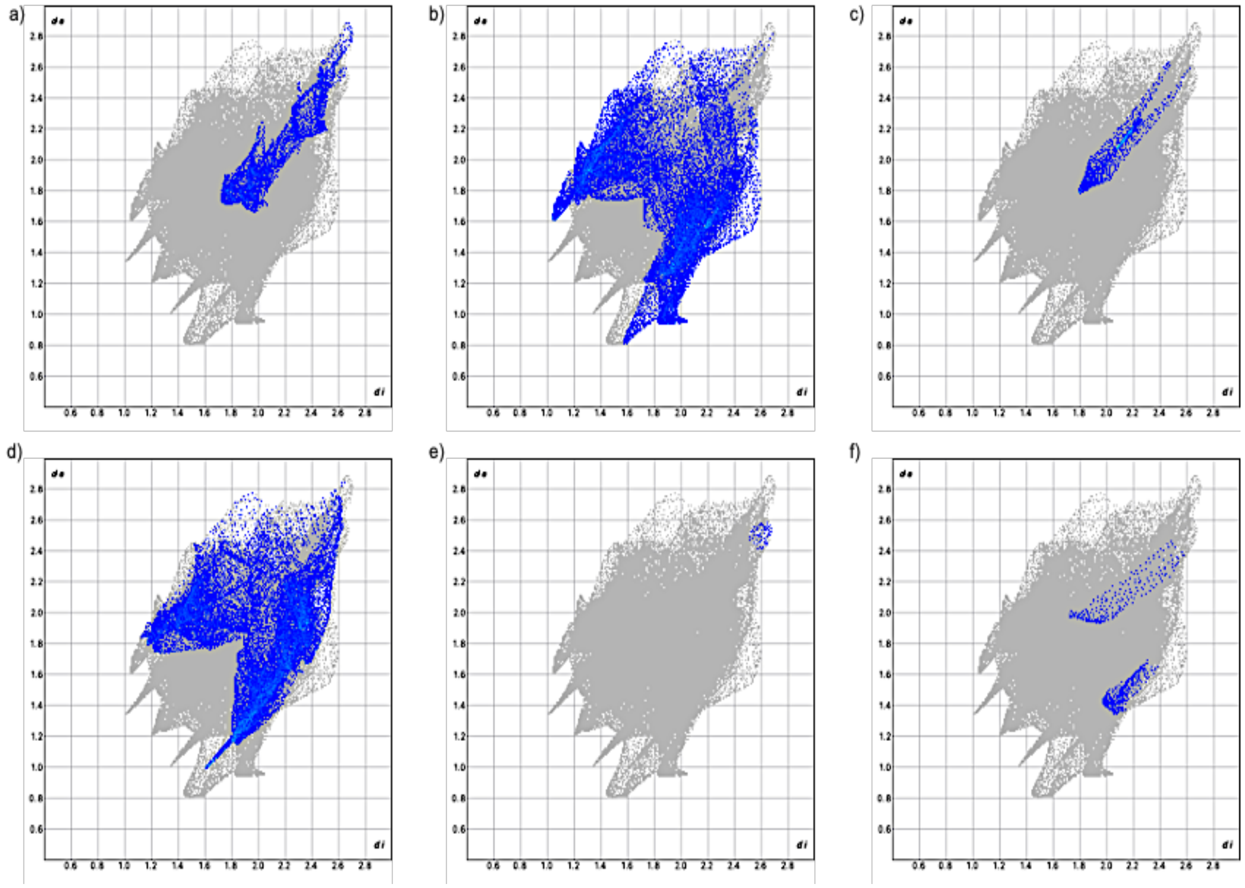

Figure S2. Selected Hirshfeld fingerprint projection interactions for **Cl-1⊃ben** structure: (a) C-C interactions, (b) C-H interactions, (c) Cl-Cl interactions, (d) Cl-H interactions, (e) Cl-N interactions, and (f) N-H interactions.

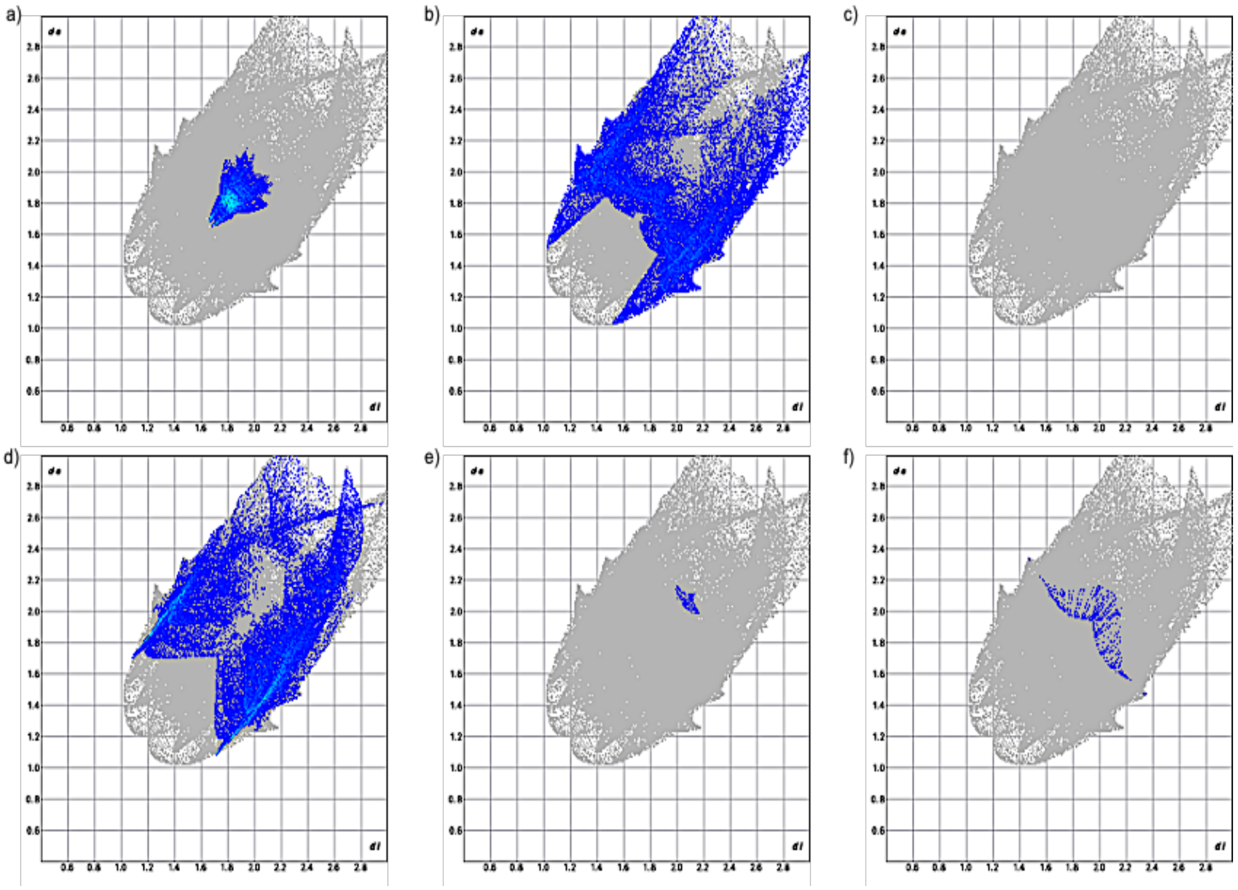

Figure S3. Selected Hirshfeld fingerprint projection interactions for **Cl-1** structure: (a) C-C interactions, (b) C-H interactions, (c) Cl-Cl interactions, (d) Cl-H interactions, (e) Cl-N interactions, and (f) N-H interactions.

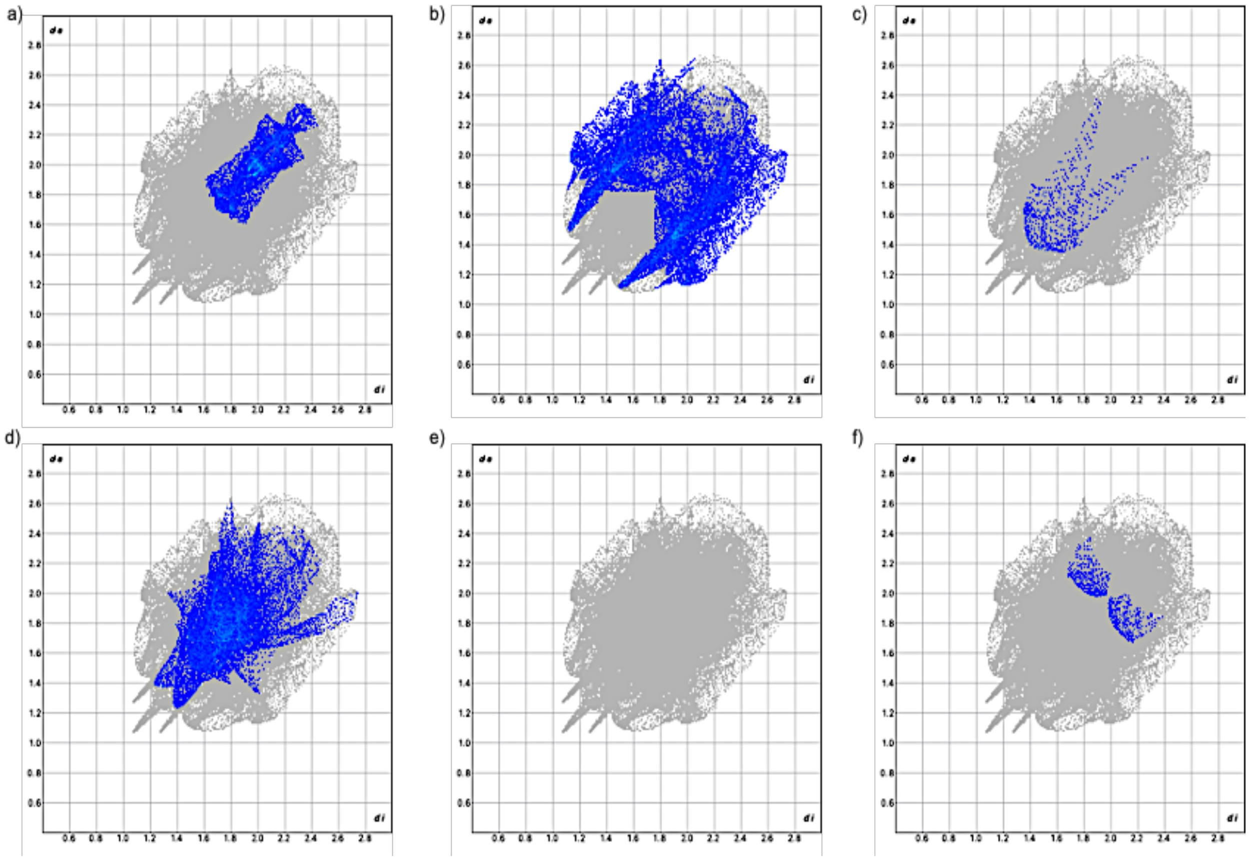

Figure S4. Selected Hirshfeld fingerprint projection interactions for **F-1** structure: (a) C-C interactions, (b) C-H interactions, (c) F-F interactions, (d) F-H interactions, (e) F-N interactions, and (f) N-H interactions.

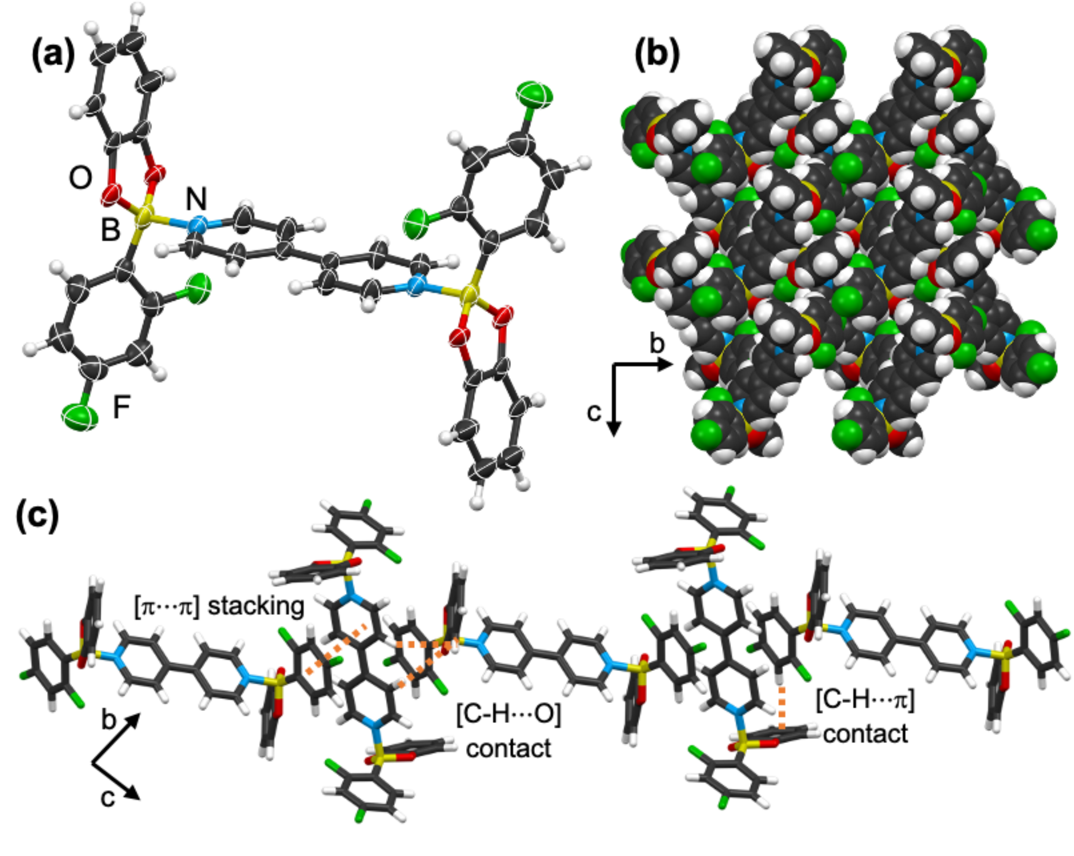

Figure S5. Single crystal X-ray structure of **F-1**: (a) Molecular unit of **F-1**, (b*)* space-filling model in the *ac*-plane*,* and (c) tapes of adjacent **FI-1** adducts supported by [C-H···O], [C-H···π] and [π···π] contacts.

S3. NMR spectral data

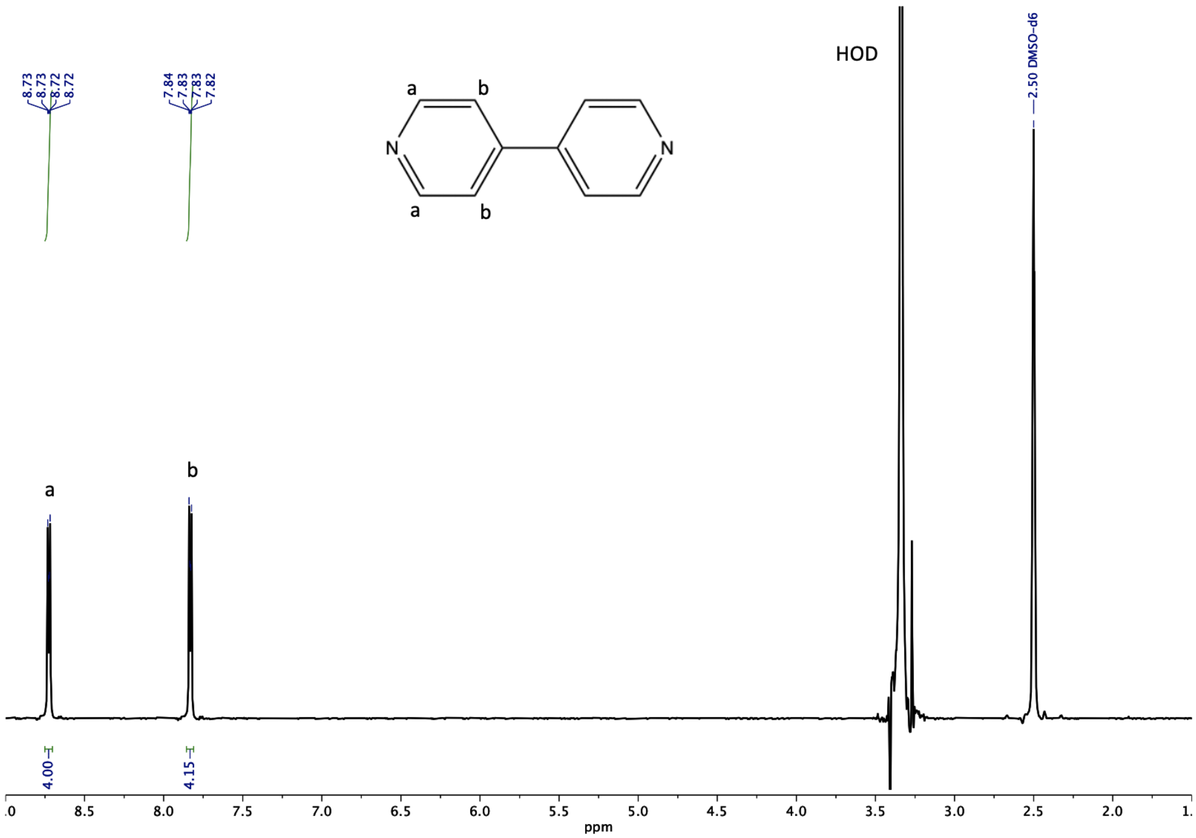

Figure S6. ^{1}H NMR spectra of **bpy** (400 MHz, DMSO-d_6).

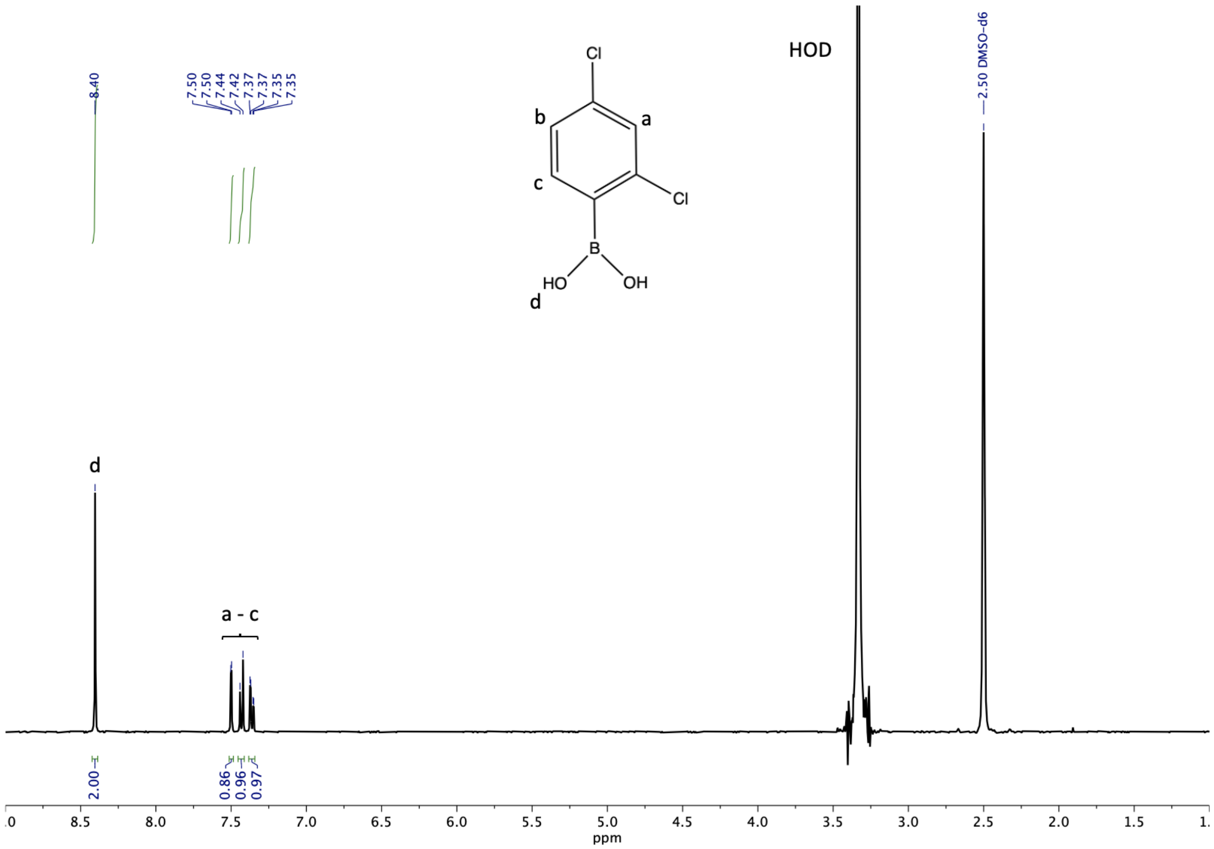

Figure S7. ^{1}H NMR spectra of **Cl-ba** (400 MHz, DMSO-d_6).

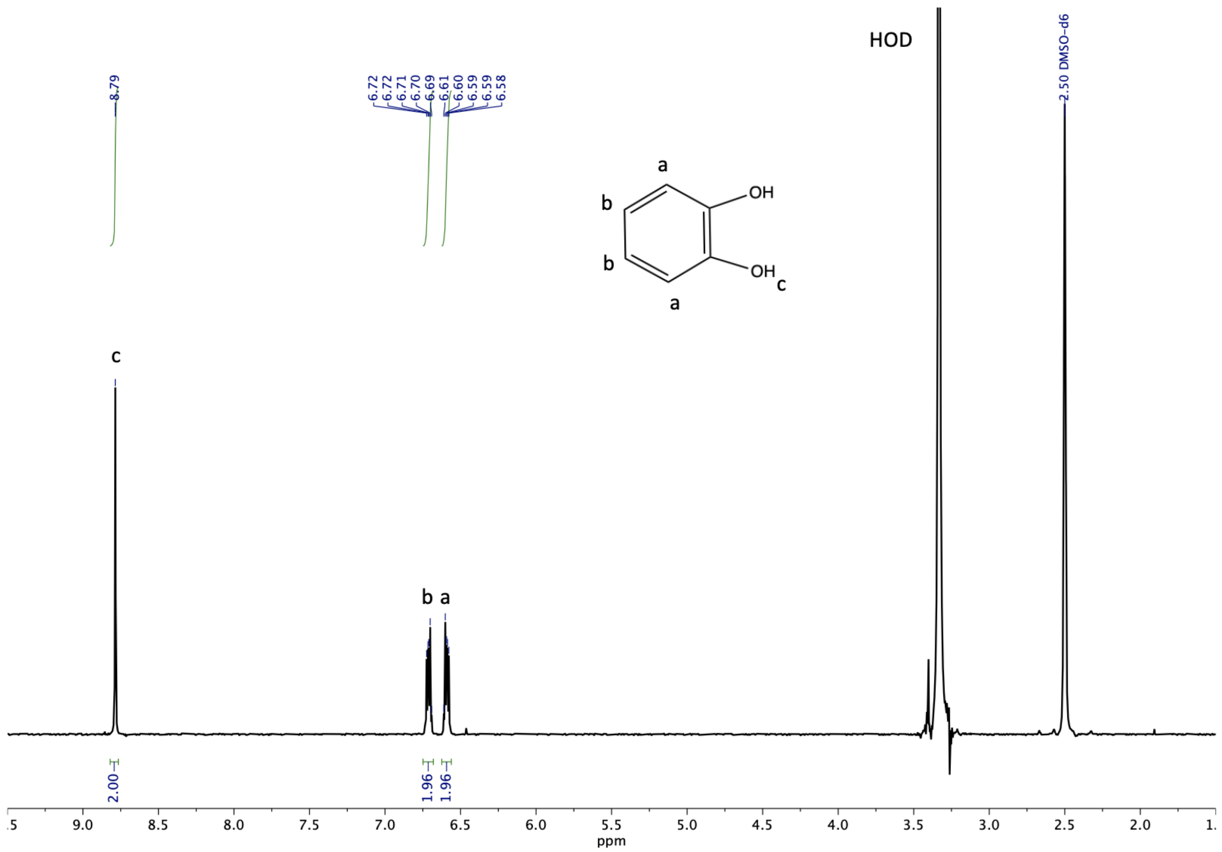

Figure S8. ^{1}H NMR spectra of **cat** (400 MHz, DMSO-d_6).

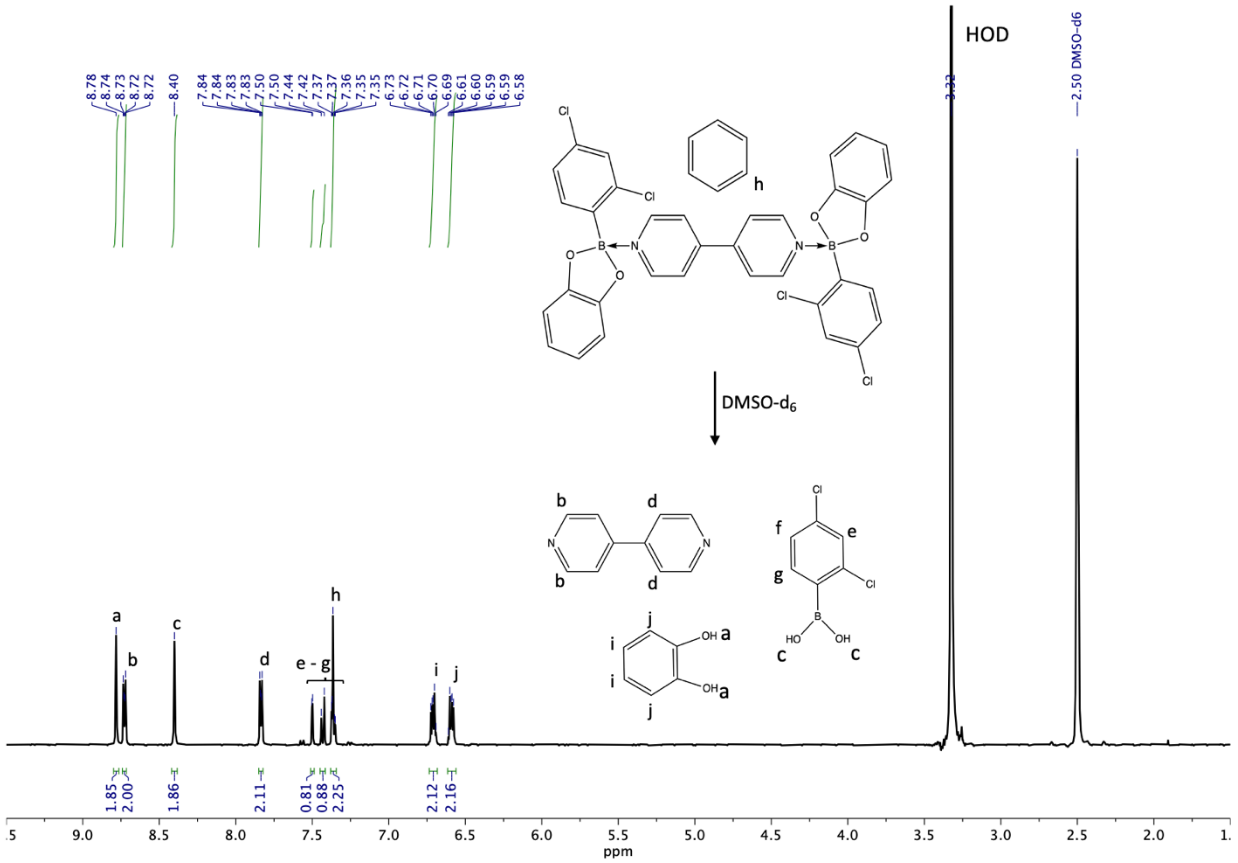

Figure S9. ^{1}H NMR spectra of **Cl-1** separation study. The signature peak of **MeCN** in DMSO-d$_6$ was not observed at 2.07 ppm[4]. The peak for **ben** in DMSO-d$_6$ was observed at 7.37 ppm[4], suggesting that only solvated adduct **Cl-1⊃ben** was generated in the separation experiment (400 MHz, DMSO-d_6).

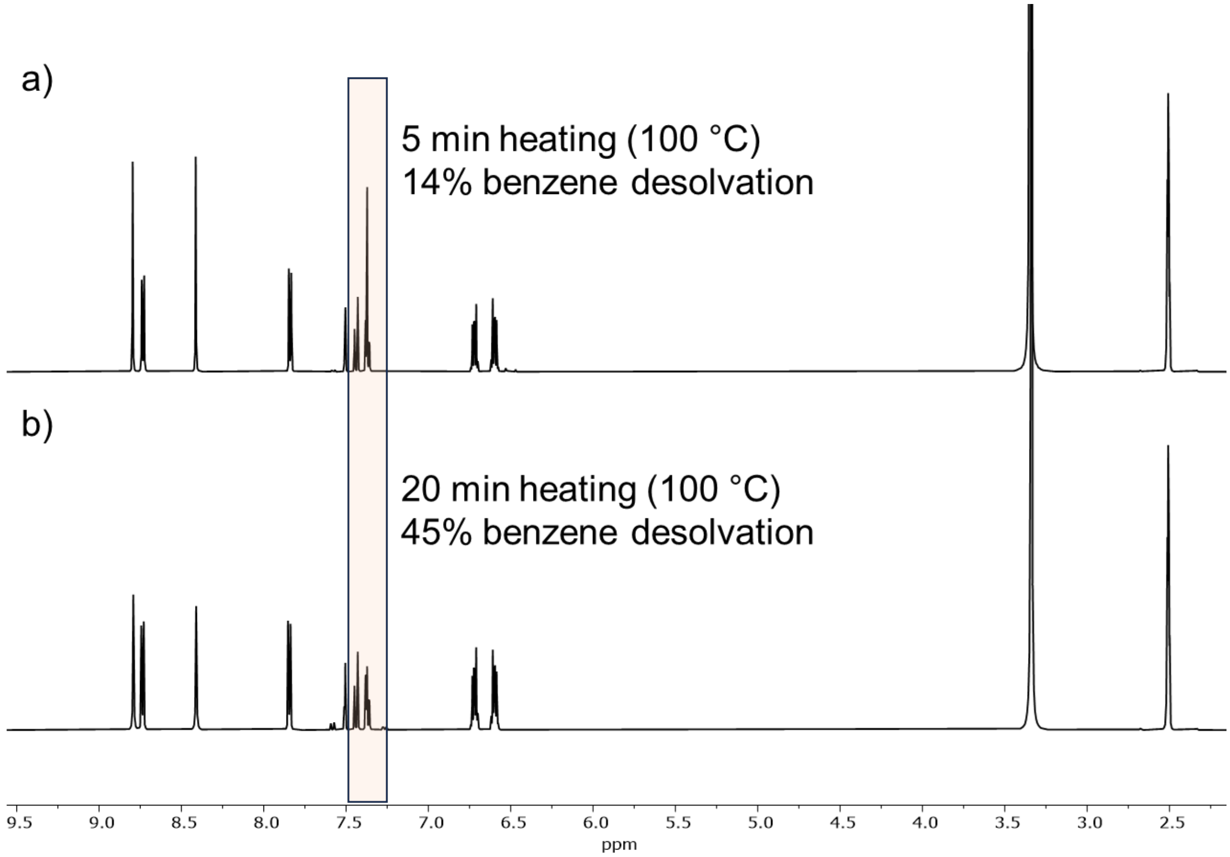

Figure S10. ^{1}H NMR spectra of desolvation experiments of **Cl-1⊃ben** crystals after heating for 100 °C for a) 5 min and b) 20 min. The peak for **ben** in DMSO-d_6 is observed at 7.37 ppm[4] (400 MHz, DMSO-d_6).

S4. Energy Framework Calculations

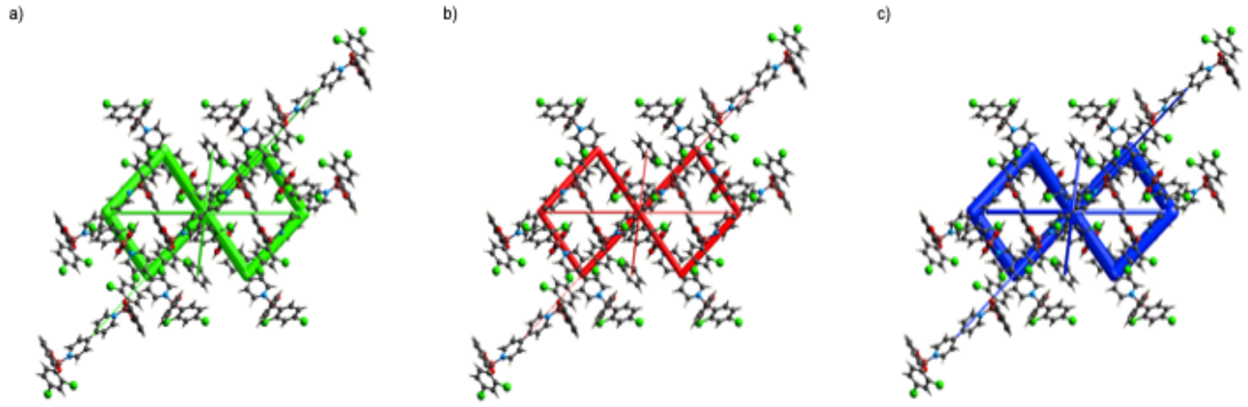

Figure S11. Energy Frameworks along the *b*-axis of **Cl-1⊃ben** calculated using CrystalExplorer software [HF/3-21G]: a) Dispersion Energy, b) Coulomb Energy, and c) Total Energy.

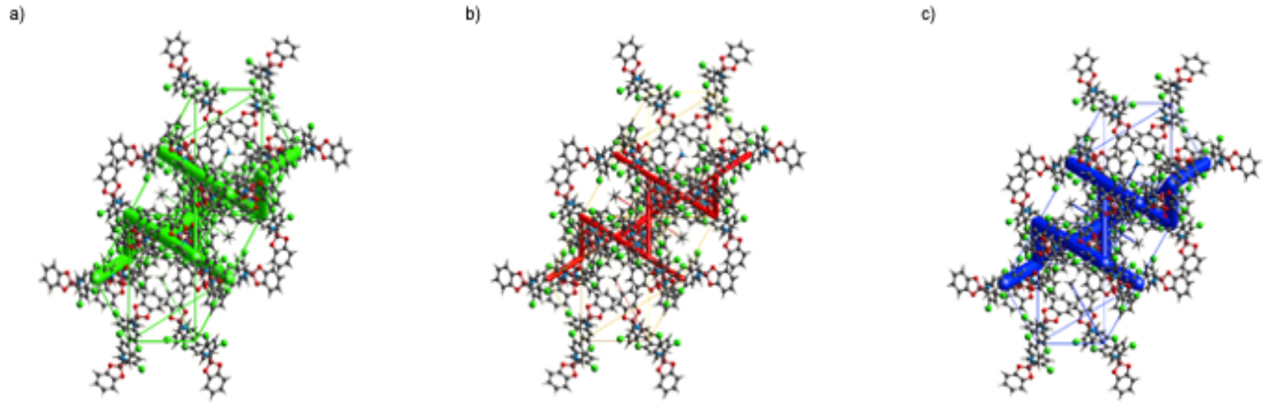

Figure S12. Energy Frameworks along the *b*-axis of **Cl-1⊃MeCN** calculated using CrystalExplorer software [HF/3-21G]: a) Dispersion Energy, b) Coulomb Energy, and c) Total Energy.

References

(1) Sheldrick, G. M. Crystal structure refinement with SHELXL. *Acta Cryst. C* **2015**, *71* (1), 3-8.

(2) Sheldrick, G. M. SHELXT–Integrated space-group and crystal-structure determination. *Acta Cryst. A* **2015**, *71* (1), 3-8.

(3) Dolomanov, O. V.; Bourhis, L. J.; Gildea, R. J.; Howard, J. A.; Puschmann, H. OLEX2: a complete structure solution, refinement and analysis program. *J. Appl. Crystallogr.* **2009**, *42* (2), 339-341.

(4) Fulmer, G. R.; Miller, A. J. M.; Sherden, N. H.; Gottlieb, H. E.; Nudelman, A.; Stoltz, B. M.; Bercaw, J. E.; Goldberg, K. I. NMR Chemical Shifts of Trace Impurities: Common Laboratory Solvents, Organics, and Gases in Deuterated Solvents Relevant to the Organometallic Chemist. *Organometallics* **2010**, *29* (9), 2176–2179.
